# The influence of perceived government corruption on depressive symptoms with social status as a moderator

**DOI:** 10.1038/s41598-022-25371-3

**Published:** 2022-12-01

**Authors:** Yujie Zhang

**Affiliations:** School of International and Public Affairs, Shanghai Jiao Tong University, Shanghai, China

**Keywords:** Psychology, Psychiatric disorders, Health policy, Public health, Quality of life, Risk factors

## Abstract

Perceived government corruption is an important indicator of depressive symptoms. Recent studies have explored the relationship between perceived government corruption and depressive symptoms in a cross-cultural context, but the underlying mechanisms need further research. This paper examines the impact of perceived government corruption on depressive symptoms in China and the moderating role of social status. Based on the 2018 wave of China Family Panel Studies (CFPS2018), 14,116 respondents aged between 16 and 96 were selected. The results revealed: (1) Perceived government corruption was significantly positively correlated with depressive symptoms. (2) Social class had an inhibitory effect in moderating the relationship between perceived government corruption and depressive symptoms. (3) The moderating effect was only significant for respondents who received education between junior high school and a bachelor’s degree. The findings provide policy implications for developing countries and transitional societies like China. To build a more psychologically healthy society, we need to strengthen anti-corruption, stimulate social mobility, and improve people's sense of gain in the future

## Introduction

Perceived government corruption is a hidden danger, which has a wide corrosive impact on society. Corruption undermines democracy and the rule of law, leads to human rights violations, distorts markets, undermines the quality of life, and allows organized crime, terrorism, and other threats to human security to flourish^1–5^. Past studies have agreed that perceived government corruption is a subjective reflection of actual corruption, which is also harmful to social development^6,7^, but the impact of perceived government corruption is the most devastating in developing countries because it diverts funds intended for development, undermines the ability of governments to provide basic services, fosters inequality and injustice, and hinders foreign investment and assistance^8,9^^.^ Perceived government corruption is a major factor in poor economic performance and a major obstacle to meeting people's needs for a better life^10^. With the deepening of people's understanding of the impact of perceived government corruption, the focus on its impact has shifted from economy to individual psychology^11^.

Perceived government corruption can exacerbate a sense of injustice and reduce trust in government, which has been confirmed by studies to exacerbate depressive symptoms^12^. Depression is a major public health issue. Globally, it is estimated that 5% of adults suffer from depression^13^. During COVID-19, the social isolation caused by the pandemic has created unprecedented pressure for depression. Related to this is that people's ability to work, seek the support of their friends, and participate in community activities is limited, resulting in loneliness, fear of infection, sadness after bereavement, and economic concerns, which exacerbates the stressors of depression^14–17^. This has sounded an alarm to all countries, making them pay more attention to mental health and better improve the mental health of their people. In this context, given that corruption is a long-standing institutional problem, which will virtually affect everyone's depressive symptoms level for a long time, new corruption-related problems may arise during the epidemic prevention period. Therefore, it is of great significance to study the mechanism of how perceived government corruption influences depressive symptoms and formulate corresponding policies.

Past studies have confirmed a positive correlation between perceived government corruption and depressive symptoms^18,19^. From a comparative perspective, a study consisting of 185 countries suggested that perceived government corruption had a negative impact on mental health and this influence was more obvious in high-income countries compared with low-income countries^20^. One rationale for this influence is that perceived government corruption will intensify social contradictions, aggravate the gap between the rich and the poor, and the original social problems cannot be solved with sufficient resources^21^. At the same time, as perceived government corruption is also a reflection of real corruption, a corrupt government ignores or infringes on the most basic survival interests of vulnerable groups and violates the original spirit of the legislation, bending the law for personal gain, and affecting people's access to basic public services^22^, thus exacerbating individual depressive symptoms. Another rationale is that perceived government corruption will hinder economic development because it can destroy fair competition and destroy trust in government^23^, thus affecting the social mobility of individuals and the allocation of social resources^24^. When individuals are difficult to achieve self-development and improve social status, they will have a sense of frustration^25^, which will exacerbate depressive symptoms. In developing countries, the society is in a period of transformation, with great social mobility and drastic changes in social status^26–28^. Thus, based on the second rationale, investigating the relationship between perceived government corruption, social status and depressive symptoms has special enlightening significance for the policy design of transitional societies.

This article is organized as follows. “Literature review” reviewed the literature on social rank theory to lay the foundation for proposing hypotheses. “Methods” presents the method, sample, and measurements. “Results” shows the main results, which include correlation analyses, hierarchical regression analyses, moderating effect analyses, heterogeneity analyses, and a robustness test. “Discussion” provides a discussion on the innovation points, limitations, and future development direction of the research findings.

## Literature review

According to the social rank theory (SRT), depressive symptoms stem from the perception of oneself being of lower social status rank than others^29^. People who see themselves as having relatively low social status tend to blame themselves for a lack of ability^30^. Moreover, the lower social status would trigger both shame and anger as people with lower social status have higher possibilities to lose in the social competition for higher salaries, favored partners, and more social resources^31^. A meta-analysis summarized 54 related studies and concluded that social status-related comparison was significantly correlated with depressive symptoms^32^. In transitional societies, as the channel for the rise of social status has not been solidified, people can often achieve the rise of social status through various ways, such as education and investment^33,34^. Moreover, due to the immature social laws and regulations, the social status is unstable and may change dramatically^35^. At present, studies focusing on transitional society have also found a negative relationship between social status and depressive symptoms^36^. and unlike in developed countries, this relationship was non-linear in developing countries like China^37^.

Past studies also suggested that there is a complex correlation between corruption and social status. According to relative deprivation theory (RD)^38^, people of different social statuses perceive government corruption differently. For instance, people with higher social status have more access to social resources and are more likely to benefit from the status quo, thus they have a lower perception of perceived government corruption^39^. Whereas people with lower social status are more sensitive to perceived government corruption because they are less able to pay the bribes needed to obtain public services. What’s more, they know little about the operation of the bureaucracy and the potential recourse when they find themselves being asked for bribes, thus, they are most likely victims of government corruption^40^. Meanwhile, some studies have demonstrated that the positive relationship between perceived government corruption and depressive symptoms may be distorted due to cultural factors^41^. Individuals in collectivist cultures may be less likely to perceive government corruption than those in individualist cultures^42^. For a country with a strong collectivist culture like China, the elite ideology that encourages people to maintain and support the current hierarchical structure and elite management have led to the recognition of government corruption^43^, which makes the relationship between perceived government corruption and social status blurred and needs to be further explored.

In contemporary China, investigating the influence of perceived government corruption on depressive symptoms with social status as a moderator has both theoretical and practical value. In 2019, the Fourth Plenary Session of the 19th Central Committee of the Communist Party (CPC) deliberated and adopted the decision of the CPC Central Committee on several major issues concerning upholding and improving the socialist system with Chinese characteristics and promoting the modernization of the national governance system and governance capacity. This makes corruption governance an important research issue of national governance theory. What’s more, with social development, the government attaches great importance to strengthening social construction, adheres to the people-centered development thought, and studies the mechanism of how perceived government corruption influences depressive symptoms is conducive to formulating relevant policies to improve people's sense of gain, happiness, and security.

To summarize the above discussion, this study proposed the following hypotheses:

Hypothesis 1: Perceived government corruption has a significant positive influence on depressive symptoms.

Hypothesis 2: Social status can significantly moderate the influence of perceived government corruption on depressive symptoms.

## Methods

### Data and sample

The data used in this study are from the 2018 wave of China Family Panel Studies (CFPS2018), which is conducted by the Institute of Social Science Survey of Peking University. The target sample size of CFPS is 16,000 households, of which 8000 households are obtained from the oversampling of five independent subsample boxes (referred to as large sample boxes for short) in Shanghai, Liaoning, Henan, Gansu, and Guangdong, and each independent subsample box has 1600 households. Another 8000 households are selected from sample boxes of 20 other provinces (short for small sample boxes). After the second sampling, the large sample boxes and the small sample boxes together constitute a national representative total sample box. The CFPS sample covers the population of 25 provinces/cities/autonomous regions in China except for Hong Kong, Macao, Taiwan, Xinjiang, Tibet, Qinghai, Inner Mongolia, Ningxia, and Hainan. The population of these 25 provinces/municipalities/autonomous regions accounts for about 95% of the total population of the country (excluding Hong Kong, Macao, and Taiwan), so the CFPS sample can be regarded as a representative sample of China.

The samples of each sample box of CFPS are extracted in three stages. The first stage sample (PSU) is the administrative district/county, the second stage sample (SSU) is the administrative village/neighborhood committee, and the third stage (terminal) sample (TSU) is the household. In the first two stages of CFPS, the official administrative division data is used for sampling. In the third stage, the map address method is used to build an end sampling box, and the cyclic equidistant sampling method with random starting points is used to sample households. Table 1 shows the sampling procedure of CFPS.

**Table 1:** CFPS three-stage sampling procedure.

Stage	Large sample boxes (Guangdong, Gansu, Liaoning, Henan)	Large sample box (Shanghai)	Small sample boxes	Total
The first stage	4 × 16 districts and counties = 64 districts and counties	32 streets (towns)	80 districts and counties	144 sample districts and counties + 32 sample streets (towns)
The second stage	64 × 4 villages and communities = 256 villages and communities	32 × 2 villages and communities = 64 villages and communities	80 × 4 villages and communities = 320 villages and communities	640 villages and communities
The third stage	640 × [28, 42] households			19,986 households

In the third stage, theoretically, 31.23 households are sampled from 640 sample frames on average, [28, 42] represents the range of households sampled from each sample frame, that is, at least 28 households are sampled from each sample frame, and at most 42 households are sampled from each sample frame.

In addition, the CFPS2018 survey was implemented by a group of trained researchers through face-to-face interviews, which ensure the high quality of the data. The actual sample size of CFPS2018 was 37,354. Information on missing variables was excluded, and this study finally included 14,116 samples in the present analyses. Most of the missing data is caused by the logic skipping in the database or the inapplicability of the respondent's identity. Moreover, in addition to the missing data due to logic skipping or inapplicability, there are also some data missing because the interviewee refused to answer, did not know, or due to some technical issues of the questionnaire access system, though the missing data size involved in technical issues is small.

Therefore, considering the missingness in this database is not completely randomly distributed across cases, that is, missing at non-random (MANR). The lack of data is not only related to the value of other variables but also related to its own value. For example, when investigating income, the data is missing because high-income people may not be willing to provide annual family income for various reasons. According to Pepinsky’s research, which compares the performance of multiple imputations and casewise deletion using a simulation approach, casewise deletion is a relatively better method^44^.

Moreover, most missing data are caused by logic skipping. Some of the missing data are since the interviewees' identities are not suitable for answering this question, which also means that the missing data cannot provide any information, and it is appropriate to delete them in column^45^. In addition, there is still a large amount of data in the database to ensure the statistical efficacy of the study, so this article used casewise deletion method (listwise deletion), which involves removing all questionnaire answers from a respondent because responses to one or more questions are missing from that respondent.

## Measures

### Perceived government corruption (independent variable)

In CFPS2018, the respondents were asked about their perception of perceived government corruption, namely, “How serious do you think the problem of perceived government corruption is in China?” The answers ranged from 0 (not at all) to 10 (very serious).

### Social status (moderating variable)

IN CFPS2018, the respondents were asked about their perception of social status, namely, “How do you rate your local social status?” The answers ranged from 1 (very low) to 5 (very high).

### Depressive symptoms (dependent variable)

Based on the Center for Epidemiologic Studies Depressive symptoms Scale (CES-D)^46^, in CFPS2018, the respondents were asked about the frequency of different feelings in the past week to assess depressive symptoms, namely, “How often do you feel depressed?” “How often do you feel it is hard to do anything?” “How often do you feel it is hard to sleep?” “How often do you feel unpleasant?” “How often do you feel lonely?” “How often do you feel unhappy?” “How often do you feel sad?” “How often do you feel life can’t go on?” The answers ranged from 1 (almost never) to 4 (almost every day). In this study, Cronbach’s $\propto$ was 0.76.

### Control variables

Based on previous studies^47–50^, this study selected sex (0 = female, 1 = male), age (continuous variable), marital status (0 = unmarried, 1 = married), education (1 = below junior high school, 2 = above junior high school, below bachelor’s degree, 3 = above bachelor’s degree) and residence registration (0 = rural, 1 = urban).

### Variable description

Table 2 displays the descriptive statistics of all variables. The mean perceived government corruption score was 6.805, and the standard deviation was 2.718, which indicates that most respondents had medium to a high level of perceived government corruption perception and that there was no large disparity within this variable. The mean social status score was 2.956, which suggests that most respondents thought their social status was fair. Meanwhile, the mean depressive symptoms score was 13.555, which shows that most respondents had relatively good mental health status. There were slightly more males than females in this study. Moreover, their age ranged from 16 to 96, which was representative of the whole population. Most respondents received junior high school education. More than half of them were married and had rural residence registration.

**Table 2:** Descriptive statistics (N = 14,116).

Variable	Mean	SD	Min	Max	N	%
Perceived government corruption	6.805	2.718	0	10	14,116	
Social status	2.956	1.024	1	5	14,116	
Depressive symptoms	13.555	3.752	8	32	14,116	
**Sex**			0	1		
Female					7015	49.700
Male					7101	50.300
Age	36.556	12.927	16	96	14,116	
**Marital status**			0	1		
Unmarried					3363	23.820
Married					10,753	76.180
Education	1.778	0.606	1	3	14,116	
**Residence registration**			0	1		
Rural					10,762	76.240
Urban					3354	23.760

### Statistical analyses

**Figure 1:**
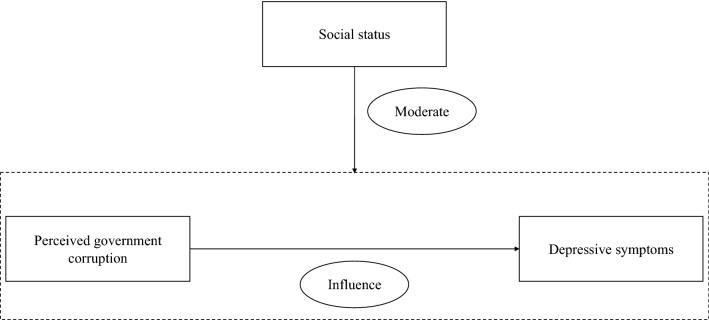
Research framework.

All the statistical analyses were performed with Stata version 16.0 (StataCorp, Texas of United States). First, this study conducted a correlation analysis among variables. Second, this study performed hierarchical regression analyses to investigate the relationship among variables and determine the possible moderating role of social status between perceived government corruption and depressive symptoms. Third, based on the OLS model, moderating effect analyses were performed to verify whether social status moderated the influence of perceived government corruption on depressive symptoms. In addition, based on education background, heterogeneity analyses were performed. Finally, this study conducted a robustness test by adding an important control variable, income status. And the results were robust. In the present study, the Variance Inflation Factor (VIF) values were < 10, which indicated that multicollinearity was not an issue in the estimate. Figure 1 presents the research framework of this study.

### Ethical approval

No ethics approval was required for this study. The data were obtained from a publicly accessible database of CFPS with a signed data use agreement.

## Results

### Correlation analyses

Table 3 displays the results of Pearson’s correlation analyses. Perceived government corruption was found to have a positive association with depressive symptoms (coefficient = 0.050, p < 0.001), although the coefficient value was small, which provided initial support for Hypothesis 1. Social status was found to have a significant correlation with both perceived government corruption and depressive symptoms, which laid the foundation to explore the role of social status between perceived government corruption and depressive symptoms.

**Table 3:** Correlation among variables (N = 14,116).

Variables	1	2	3	4	5	6	7	8
1. Perceived government corruption	1.000							
2. Social status	−0.074***	1.000						
3. Depressive symptoms	0.050***	−0.119***	1.000					
4. Sex	0.030***	−0.016	−0.057***	1.000				
5. Age	−0.061***	0.179***	0.080***	0.000	1.000			
6. Marital status	0.053***	0.076***	−0.038***	−0.114***	0.292***	1.000		
7. Education	0.096***	−0.102***	−0.125***	0.034***	−0.477***	−0.146***	1.000	
8. Residence registration	0.041***	-0.035***	-0.068***	-0.003	0.003	-0.014	0.340***	1.000

***p < 0.001, **p < 0.01, *p < 0.05.

### Hierarchical regression analyses

Table 4 displays the results of hierarchical regression analyses. In the first step, perceived government corruption was found to be positively correlated with depressive symptoms (coefficient = 0.098, p < 0.001). Control variables, including sex, marital status, and education significantly explained depressive symptoms in a negative way, while age and residence registration positively explained depressive symptoms. In the second step, perceived government corruption was positively correlated with depressive symptoms (coefficient = 0.086, p < 0.001). Social status was negatively correlated with depressive symptoms (coefficient = −0.450, p < 0.001). Control variables explained depressive symptoms in the same way as which in Step 1. In the third step, perceived government corruption was positively correlated with depressive symptoms (coefficient = 0.181, p < 0.001). Social status was negatively correlated with depressive symptoms (coefficient = −0.283, p < 0.001). Interaction of perceived government corruption and social status was negatively correlated with depressive symptoms (coefficient = −0.032, p < 0.001). Control variables explained depressive symptoms in the same way as which in Step 1 and Step 2. It was suggested that social status may play a moderating role in the association of perceived government corruption and depressive symptoms, thus, further investigation should be conducted. In sum, the results supported Hypothesis 1.

**Table 4:** Hierarchical regression results (N = 14,116).

Variable	Step 1 (DV: depressive symptoms)	Step 2 (DV: depressive symptoms)	Step 3 (DV: depressive symptoms)
Perceived government corruption	0.098*** (8.47)	0.086*** (7.49)	0.181*** (5.77)
Social status		−0.450*** (−16.28)	−0.283*** (−3.85)
Perceived government corruption × social status			−0.032*** (−3.26)
Sex	−0.492*** (−7.83)	−0.503*** (−8.09)	−0.505*** (−8.12)
Age	0.018*** (6.15)	0.024*** (8.43)	0.024*** (8.31)
Marital status	−0.725*** (−9.40)	−0.690*** (−9.02)	−0.690*** (−9.03)
Education	−0.611*** (−9.61)	−0.611*** (−9.71)	−0.616*** (−9.78)
Residence registration	0.343*** (−4.32)	−0.382*** (−4.85)	−0.379*** (−4.82)

t-values in parentheses, ***p < 0.001, **p < 0.01, *p < 0.05.

To better understand the potential moderating role of social status, it is necessary to understand the influence of social status on perceived government corruption first. Table 5 shows that social status was significantly negatively related to perceived government corruption (coefficient = −0.172, p < 0.001). Controls variables such as sex, marital status and education were significant and positive predictors of perceived government corruption, while age was negatively related to perceived government corruption. In sum, individuals with higher social status perceived less government corruption.

**Table 5:** The influence of social status on perceived government corruption (N = 14,116).

Variable	Coefficient	t-value	p >|t|
Social status	−0.172***	−7.63	0.000
Sex	0.195***	4.26	0.000
Age	−0.008***	−3.67	0.000
Marital status	0.538***	9.61	0.000
Education	0.350***	7.58	0.000
Residence registration	0.087	1.51	0.131

***p < 0.001, **p < 0.01, *p < 0.05.

### Moderating effect analyses

Table 6 illustrates the moderating role of social status. After the centralization of perceived government corruption and social status, the regression results revealed that social status played an inhibitory role in moderating the influence of perceived government corruption on depressive symptoms (coefficient = −0.032, p < 0.001). Moreover, age was significantly positively related to depressive symptoms, while sex, marital status, education, and residence registration were significantly negatively related to depressive symptoms.

**Table 6:** Moderating effect results (N = 14,116).

Variable	Coefficient	t-value	p >|t|
C_Perceived government corruption	0.087***	7.62	0.000
C_Social status	−0.499***	−16.27	0.000
C_Perceived government corruption × C_ Social status	−0.032***	−3.26	0.001
Sex	−0.505***	−8.12	0.000
Age	0.024***	8.31	0.000
Marital status	−0.690***	−9.03	0.000
Education	−0.616	−9.78	0.000
Residence registration	−0.379***	−4.82	0.000

***p < 0.001, **p < 0.01, *p < 0.05.

**Figure 2:**
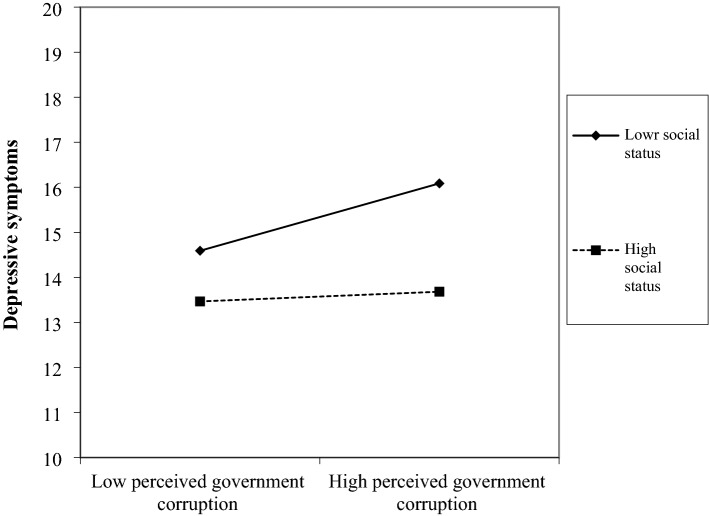
The moderating role of social status. High or low levels of perceived government corruption are to add or subtract one standard deviation from the average value of perceived government corruption. High or low levels of social status are to add or subtract one standard deviation from the average value of social status.

Figure 2 plots the interaction of perceived government corruption and social status. It was suggested that social status had a strong inhibitory effect on the influence of perceived government corruption on depressive symptoms. Perceived government corruption was positively correlated with depressive symptoms. Compared with the low social status situation, the regression slope of individual depressive symptoms in the high social status situation was not steeper. This shows that the negative impact of perceived government corruption on depressive symptoms was weakened in the context of high social status. In addition, it can be seen from Fig. 2 that for individuals with high perceived government corruption, low social status will also aggravate their depressive symptoms. Therefore, Hypothesis 2 is further verified.

Table 7 shows the results of simple slope tests. The output in Table 7 were simple slopes (computed at mean + 1sd, mean, and mean − 1sd of the moderator) for the effect of perceived government corruption on depressive symptoms at three relative levels of social status. The dy/dx column contains the simple slopes at three different levels of the moderator. Three slopes were positive and statistically significant (with p’s < 0.001). Notably, the slopes do appear to become steeper as the value moves from high levels of social status (the moderator) to low levels of social status (the moderator). As can be seen, 0.055 < 0.087 < 0.120. This also means that the lower the social status, the greater the negative impact of perceived government corruption on depressive symptoms, which again confirms Hypothesis 2.

**Table 7:** Simple slope tests results (N = 14,116).

Value of social status (moderator)	dy/dx	Standard error	t-value	p >|t|
Mean + 1sd = 3.980	0.055***	0.015	3.68	0.000
Mean = 2.956	0.087***	0.011	7.62	0.000
Mean − 1sd = 1.933	0.120***	0.016	7.73	0.001

***p < 0.001, **p < 0.01, *p < 0.05.

### Heterogeneity analyses based on education

Table 8 shows the results of heterogeneity analyses. For respondents who received below junior high school education and received above bachelor’s degree education, the moderating effect of social status was insignificant. Whereas respondents received education between junior high school and bachelor’s degree, social status had a significant inhibitory effect on the association between perceived government corruption and depressive symptoms. Notably, all control variables were significant for respondents who received below bachelor’s degree education. While respondents received above bachelor’s degree education, only age was a significant and positive predictor of depressive symptoms.

**Table 8:** Heterogeneity analyses results (N = 14,116).

Variable	By education		
	Below junior high school (N = 4502)	Above junior high school, below bachelor’s degree (N = 8242)	Above bachelor’s degree (N = 1372)
C_Perceived government corruption	0.090*** (4.23)	0.088*** (6.03)	0.049 (1.31)
C_Social status	−0.371*** (−6.84)	−0.563*** (−13.99)	−0.899*** (−8.15)
C_Perceived government corruption × C_ Social status	−0.013 (−0.85)	−0.047*** (−3.40)	−0.011 (−0.25)
Sex	−0.774*** (−6.12)	−0.422*** (−5.53)	−0.161 (−0.94)
Age	0.017*** (4.08)	0.021*** (4.20)	0.047** (2.60)
Marital status	−1.430*** (−8.26)	−0.500*** (−5.05)	−0.285 (−1.36)
Residence registration	−0.872*** (−3.92)	−0.416*** (−4.70)	0.043 (0.23)

t-values in parentheses, ***p < 0.01, **p < 0.05, *p < 0.1.

### Robustness test

Adding a vital control variable is one of the effective means for the robustness test. This study added income status, namely, “How would you rate your local income status?” for the robustness test. The answers ranged from 1 (very low) to 5 (very high). If the conclusions of the two models are basically the same, the conclusion is robust and reliable. Table 9 shows that after a new control variable was added to run a regression analysis, the results were consistent with previous regression models, so the results were robust.

**Table 9:** Robustness test results (N = 14,116).

Variable	Coefficient	t-value	p >|t|
C_Perceived government corruption	0.085***	7.42	0.000
C_Social status	−0.343***	−9.57	0.000
C_Perceived government corruption × C_Social status	−0.029**	−3.00	0.003
Sex	−0.495***	−7.98	0.000
Age	0.023***	8.05	0.000
Marital status	−0.668***	−8.75	0.000
Education	−0.612***	−9.74	0.000
Residence registration	−0.378***	−4.82	0.000
Income status	−0.299	−8.39	0.000

***p < 0.01, **p < 0.05, *p < 0.1.

## Discussion

Corruption and depressive symptoms are both global governance issues, and the investigation of the nuanced mechanisms linking perceived government corruption and depressive symptoms is conducive to promoting a healthier and more equal society worldwide. Consistent with the hypotheses, the results showed that perceived government corruption was positively correlated with depressive symptoms. At the same time, social status had a strong inhibitory effect on the association between perceived government corruption and depressive symptoms. Therefore, the current findings supported Hypothesis 1 and Hypothesis 2, which implies that it is necessary to further strengthen anti-corruption efforts. It is worth noting that in the correlation analyses, perceived government corruption was found to have a positive association with depressive symptoms (coefficient = 0.050, p < 0.001), although the coefficient value was small, which still provided initial support for Hypothesis 1. At the same time, in a transitional society such as China, we should promote social mobility and social equity, generally improve people's social status, and reduce the impact of perceived government corruption on depressive symptoms. The results have proved that in a collectivist cultural country like China, the relationship between social status and perceived government corruption is different from in individualistic cultural countries. It is a negative relationship, that is, the higher the social status, the lower the perceived government corruption.

This study confirmed the positive relationship between perceived government corruption and depressive symptoms, which was in line with many previous studies^51–53^. However, this study extended the research scope to developing countries and collectivist cultural countries. A study using samples from 126 countries suggested that the impact of perceived government corruption on subjective happiness was only significant in democratic or high-income countries^54^. Subjective well-being and depressive symptoms are like two sides of a coin. This study testified that the influence of perceived government corruption on depressive symptoms was also significant in collectivistic and middle-income countries.

A previous study has found that perceived government corruption will be transmitted through individuals' trust in government and masked by online political news consumption, thus impacting depressive symptoms^55^. This finding has strengthened the understanding of the mechanism of how perceived government corruption influences depressive symptoms from the perspective of political psychology. However, the previous research has not formed a broader dialogue with sociological theories. It is worth noting that China is a transitional society. To grasp the influence of perceived government corruption on depressive symptoms from the macro perspective of social transformation, it is necessary to explore this influence from the sociological perspective of social stratification and class mobility. Mediation and regulatory variables are the third parties in the causal mechanism. At present, the mediation mechanism of perceived government corruption affecting depressive symptoms has been known. Further from the deep social structure, it is particularly important to consider the regulatory mechanism of perceived government corruption affecting depressive symptoms.

Based on social rank theory and relative deprivation theory, this study investigated the moderating role of social status between perceived government corruption and depressive symptoms. The findings suggested that social status could inhibit the influence of perceived government corruption on depressive symptoms. Meanwhile, social status was significantly negatively related to perceived government corruption. Thus, higher social status individuals perceive less government corruption and therefore have fewer depressive symptoms. This conclusion extended social rank and relative deprivation theories^56,57^. In a collectivist society, high social status is usually not the result of one's own efforts. The acquisition of social status often needs to unite families and friends to form a joint force^58^. For example, China's public ownership and collective economy are dominant^59^. While making collective profits, government corruption may also be encountered. However, thanks to the enhancement of the collective ability to obtain social resources, individuals have a higher tolerance for government corruption^60^. Therefore, considering cultural factors, on the one hand, we need to generally promote social equity and improve people's social status, on the other hand, we should also consider cultural factors and formulate measures according to local conditions.

Moreover, heterogeneity analyses based on education reflected the fact that the moderating effect of social status was only significant for people who received education between junior high school and bachelor’s degree, but insignificant for people who received education below junior high school and above bachelor’s degree. The rationale behind this phenomenon could be that in Chinese society, education is the most important channel of social mobility^61^. If people do not have a junior high school education, they are likely to be at a low-income level in society, and it is very difficult to improve their social status^62^. Moreover, these people are very sensitive to the perception of government corruption and are more vulnerable to depressive symptoms caused by social injustice^63^. If they have a bachelor's degree, they can achieve normal social mobility and are more likely to obtain a high income by relying on their own ability^64^. Moreover, they have a high tolerance for government corruption^65^. Therefore, the regulatory role of social status promotion is not obvious. For people with moderate education, the social mobility they face has great uncertainty, and the tolerance for government corruption will change with the change of their social status^66^, so they are most likely to feel the regulatory role of social status.

This study has strengths as well as some limitations. The study’s main strength was the large sample size and national-level representation of the Chinese population. Another strength is that this study was likely the first to construct a moderation model to detect the underlying mechanism of how perceived government corruption influences depressive symptoms. A limitation is that the results were cross-sectional; thus, longitudinal, and experimental studies should be conducted to reveal more causal relationships among the above variables. However, the author believes that the findings are nevertheless of value. Despite this limitation, the author feels that these results have policy value. This study contributes to the development of public and social policies for corruption governance and mental health promotion. With an emphasis on the moderating role of social status, public and social policies should pay more attention to promoting social equity and social mobility, thus improving people’s mental health in a transitional society.

The results of this study are of great significance for linking corruption governance to the field of social psychology. Paying attention to the mitigating effect of social status is particularly important for the social effect of anti-corruption. On the one hand, in the society of collectivist culture, we should pay attention to the role of social status in people's mental health. On the other hand, when people with high social status think that corruption has little impact on mental health, the government should pay attention to the ordinary majority, because they are the biggest victims of government corruption. Public policies should consider the negative effect of government corruption on the ordinary majority and promote social mobility.

## Data Availability

The CFPS datasets are publicly available at the Peking University Open Research Data platform (https://doi.org/10.18170/DVN/45LCSO). Researchers can obtain these data after submitting a data use agreement to the CFPS team.
